# The complete mitochondrial genome of *Carpodacus pulcherrimus* (Passeriformes: Fringillidae)

**DOI:** 10.1080/23802359.2020.1791007

**Published:** 2020-07-27

**Authors:** Nan Yang, Feng-jun Li, Kong Yang, Lu Qiao, Bi-song Yue

**Affiliations:** a Institute of Qinghai-Tibetan Plateau, Southwest Minzu University, Chengdu, P. R. China; b College of Life Sciences, Sichuan University, Chengdu, P. R. China

**Keywords:** *Carpodacus pulcherrimus*, complete mitochondrial genome, evolutionary relationships

## Abstract

The Himalayan Beautiful Rosefinch *Carpodacus pulcherrimus*, belongs to the family Fringillidae, distributed in central Himalayas from India (Himachal Pradesh) to southwest China and Bhutan. The conservation status of this species is least concern (LC) in IUCN. In this study, the complete mitogenome of *C. pulcherrimus* was determined. The mitogenome is a circular molecule of 16,797 bp in length, containing 13 protein-coding genes, 2 ribosome RNA genes, 22 transfer RNA genes, and 1 non-coding region. We reconstructed a phylogenetic tree based on Bayesian inference for other 14 Fringillidae species. The new mitogenome data would provide useful information for application in conservation genetics and further clarify phylogenetic evolution of this species.

The Himalayan Beautiful Rosefinch *Carpodacus pulcherrimus*, belongs to the family Fringillidae, distributed in central Himalayas from India to southwest Asia (Dickinson 2003). It is found in Bhutan, China, India, Mongolia, Nepal, and Pakistan. The species is associated with the montane and submontane forest edges, scrub, along or above tree-line, also on sparsely vegetated slopes, valleys, and steep hillsides. The species breeds at 3600–4500 m, locally to 4650 m in China. In non-breeding season found in similar habitat at lower altitude (Zhao 1995; Zheng 2011). Finches (Fringillidae) are mostly colorful, so that species limits have been rarely under debate (Collar and Newton 2010). The conservation status of this species is least concern (LC) in IUCN. In China, the species also has been listed as a LC species by the red list of China’s vertebrates (Jiang et al. 2016). Up to now, no any complete mitochondrial genome data of *C. pulcherrimus* is available in the GenBank. In this study, we sequenced the complete mitochondrial genome of *C. pulcherrimus* (GenBank number: MT648821) examined its phylogenetic position with other Fringillidae species.

The tissue samples were obtained in Chaqing Songduo Nature Reserve, Baiyu County, Sichuan Province, P. R. China (Latitude: 30.984°N, Longitude: 99.342°E, Altitude: 3808 m), and maintained in Sichuan University, Chengdu. The stored number of the sample is CQSD-015. Total genomic DNA was extracted from muscle tissue using the DNA extraction kit (Aidlab Biotech, Beijing, China). The mitochondrial genomes of *C. roseus* (KM078779.1) is used to design primers for polymerase chain reaction (PCR) and used as template for gene annotation.

The total complete mitogenome sequence of *C. pulcherrimus* is 16,797 bp, which is composed of 13 protein-coding genes (PCGs), 2 ribosome RNA genes, 22 transfer RNA genes, and 1 non-coding region (D-Loop). The total base composition of the *C. pulcherrimus* mt genome is an A + T-rich pattern of the vertebrate mitochondrial genomes. ATG is the most common start codon, ATA is used for ND3. *C. pulcherrimus* had one non-coding region: a 1225 bp control region (D-Loop).

The phylogenetic relationship for the mitochondrial genome sequences newly determined was examined with 15 Fringillidae species. The BI analysis was performed using BEAST version 1.7 (Drummond et al. 2012), and the best-fit model (GTR + I+G) of nucleotide evolution was selected using the AIC test in JModelTest 2 (Darriba et al. 2012). Phylogenetic tree resulting from the Bayesian inference (BI) analyses showed that *C. pulcherrimus* was a sister relationship to *C. roseus* (posterior probability = 1.00) (Figure 1).

**Figure 1.**
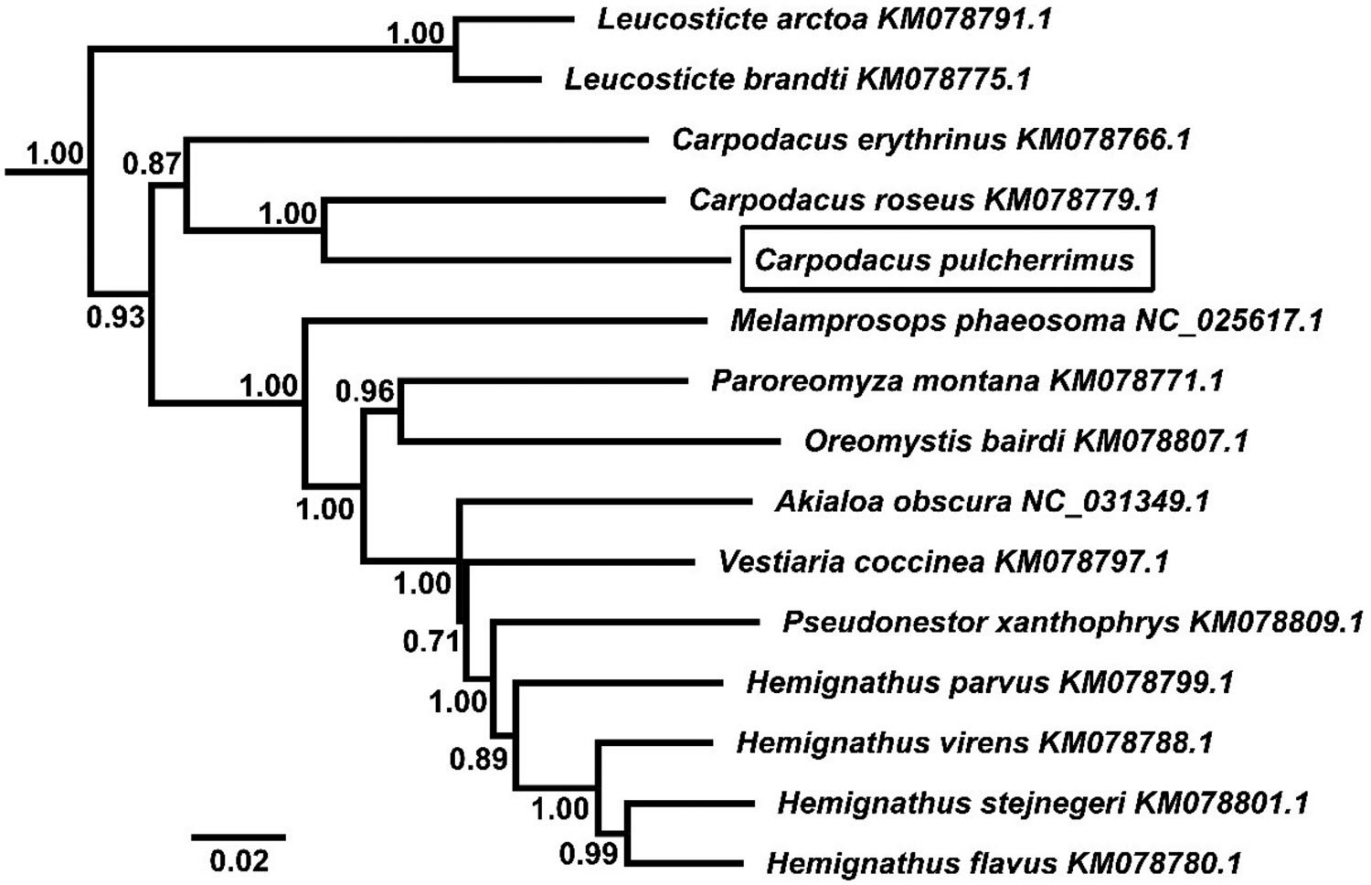
Phylogenetic tree derived from 12 protein-coding gene sequences from 15 complete mitochondrial genomes using BI analysis. Numbers by the nodes indicate Bayesian posterior probabilities.

We first report and analyze the complete mitochondrial genome of *C. pulcherrimus*. The data will contribute to solve the phylogenetic relations of the genus *Carpodacus*. And our data would provide reference information for further study of this species and serve as molecular tools to protect it.

## Data Availability

The data that support the findings of this study are openly available in GenBank of NCBI at https://www.ncbi.nlm.nih.gov, reference number MT648821.
